# The cost-effectiveness of unilateral cochlear implantation in the Finnish health care

**DOI:** 10.1007/s00405-025-09468-9

**Published:** 2025-06-06

**Authors:** Aarno Dietz, Elizabeth Seil, Pia Linder, Ismo Linnosmaa, Bonny Parkinson, Henry Cutler

**Affiliations:** 1 Department of Otorhinolaryngology, Kuopio University Hospital, P.O. Box 100, Kuopio, 70211 Finland; 2 Institute of Clinical Medicine, School of Medicine, Faculty of Health Sciences, University of Eastern Finland, Kuopio, Finland; 3 Macquarie University Centre for the Health Economy, Macquarie Business School, Sydney, Australia; 4 Department of Health and Social Management, Faculty of Social Sciences and Business Studies, University of Eastern Finland, Kuopio, Finland; 5 Welfare State Research, Finnish Institute for Health and Welfare, Helsinki, Finland; 6 Australian Institute of Health Innovation, Macquarie University, Sydney, Australia

**Keywords:** Cochlear implant, Hearing loss, Cost-effectiveness, Cost-utility, Finland

## Abstract

**Purpose:**

This study estimates the cost-effectiveness of unilateral cochlear implants (UCIs) for Finnish adults with severe to profound hearing loss within the contemporary Finnish healthcare system.

**Methods:**

We conducted a cost-utility analysis using a Markov model to compare UCIs with hearing aids for adults with severe to profound hearing loss. We developed an average clinical pathway based on data from three Finnish university hospitals to estimate resource use. The model captured health-related quality of life, potential adverse events, device upgrades, and device failure. We estimated unit costs from national expert opinions and from grey and published literature. We performed a probabilistic sensitivity analysis to evaluate how uncertain model inputs affect the incremental cost-effectiveness ratio (ICER).

**Results:**

The ICER for UCIs compared to hearing aids in the Finnish adult population was €14,528 per quality-adjusted life year (QALY). The cost-effectiveness acceptability curve indicated a 98,5% likelihood that UCIs are cost-effective compared to a €25,000 per QALY gained threshold. The ICER was most sensitive to the discount rate, UCI device and surgery costs, the utility increment from UCI treatment, costs associated with UCI fitting, and the cost and upgrade cycle of sound processors.

**Conclusion:**

UCIs for adults are cost-effective within the Finnish healthcare setting. Our results align with ICER findings for UCIs in the UK and Swedish adult populations, despite differences in indications, clinical pathways, and device unit costs. All studies suggest that UCIs are more cost-effective when implemented at an earlier age, given the same level of hearing performance.

**Supplementary Information:**

The online version contains supplementary material available at 10.1007/s00405-025-09468-9.

## Introduction

Finland is one of the most rapidly ageing societies among OECD countries [1]. While the share of individuals aged 65 years and older was 15% in the year 2000, it increased to 23% in 2020 and it is projected to increase to 26% by the year 2030 and to 29% by 2060 [2]. Like other countries in the Nordics, Finland enjoys one of the world’s most advanced and comprehensive welfare systems in which constitutions define the basic and equal economic, social and educational rights of citizens. The specific and relatively abundant entitlements of citizens are indicated through welfare legislation, which also demonstrates the statutory service duties of national and regional governments [3]. Among them is the “Act on Supporting the Functional Capacity of the Older Population and on Social and Health Care Services for Older Persons”, which entitles older persons to receive social and health care services in accordance with their individual needs. Other objectives are to support the well-being, health, functional capacity and independent living of the ageing population and to improve their opportunities for societal participation [4]. The pronounced demographic shift towards an aging population will increase the proportion of economically dependent individuals in many countries, leading to greater constraints on healthcare budgets. Therefore, implementing decision-analytic models for evidence-based assessments of the clinical and economic outcomes of health interventions will be crucial for informed decision-making, efficient healthcare resource allocation, and sustainable healthcare budgets.

Age-related hearing loss (HL) is also a growing concern in Finland’s ageing society, impacting mental well-being, social participation, and cognitive health. Untreated or inadequately treated HL is linked to depression, social isolation, and an increased risk of dementia. Cochlear implants (CIs) have effectively treated severe-to-profound HL for decades. However, there is significant under-provision of CIs especially among older adults. Main barriers include healthcare providers’ lack of awareness and the perceived high costs [5, 6]. Additionally, especially elderly patients may refrain from treatment due to fear of anesthetic and surgical complications [7].

Most health economic research for pharmaceuticals and medical devices seeks to undertake a cost utility analysis. Health outcomes are represented using the Quality-Adjusted Life Year (QALY). A QALY is estimated by weighting a life-year with a health state utility score, which is a preference-based measure of the quality of life using values between 0 and 1; 0 corresponds to death and 1 to perfect health. Thus, QALYs combine length of life and quality of life adjusted for preferences of each outcome into one measure.

The metric for cost-utility analyses is the Incremental Cost-Effectiveness Ratio (ICER), which measures the incremental cost of the intervention in comparison to alternative treatment (comparator) divided by the incremental QALYs of the intervention [8]. In some European countries, cost-effectiveness thresholds are applied for pharmacological and medical device interventions. In the Finnish health care system, no generally accepted cost-effectiveness threshold exists. However, reference is often made to the UK National Institute for Health and Care Excellence (NICE) cost-effectiveness threshold of £20,000 to £30,000/QALY (approx. €23,000 to €35,000/QALY). Higher thresholds have been applied to, e.g., pediatric interventions of rare diseases or in situations where no other treatment options are available reflecting the willingness of countries such as the UK to depart from opportunity cost principles [9–11]. ICERs do not capture all manifold factors related to the intervention or targeted population, thus cost-effectiveness should not be the only decision-making criterion [12].

The first economic evaluations of cochlear implantation were made for pediatric populations with an emphasis on speech and language development and their effect on educational and vocational prognosis [13]. Since then, cochlear implantation has evolved to include adult patients. Today, patients with age-related hearing loss constitute the most prevalent group eligible for cochlear implantation [14]. Likewise, cochlear implant surgery has evolved considerably, becoming increasingly safer and can be performed easily under local anesthesia [7]. This option has practically removed age limits and many comorbidity obstacles for providing CI treatment [7].

Only limited data on the cost-effectiveness of cochlear implantation in older adults are available. Although there are inconsistencies in study design and methodology, research examining ICERs has indicated that unilateral cochlear implantation (UCI) is a cost-effective treatment option for adults and elderly patients [15, 16]. This is particularly relevant for health care funders due to the high prevalence of age-related hearing loss in aging societies, and the costs associated with cochlear implantation. Furthermore, the increasing demand for this treatment modality should also be taken into consideration.

The primary aim of this study was to evaluate the cost-effectiveness of UCI in adults within the government-funded Finnish health care setting. The secondary aim was to compare the cost-effectiveness of UCIs between Finland, Sweden, and the UK and to investigate how different clinical pathways and unit costs across these countries affect cost-effectiveness.

## Methods

A Markov model was employed to evaluate the cost-effectiveness of UCIs in the eligible Finnish adult population with hearing-aids. Results were compared to two other studies that used the same methods and model to evaluate the cost-effectiveness of UCIs in the UK and Sweden, thereby removing the potential for results to differ due to different methods and model structures [15, 16].

The model captured the chronic nature of severe to profound hearing loss in adults, treatment pathways, and health outcomes associated with UCIs and hearing-aids. The model adopted a Finnish health care system perspective and a lifetime horizon to capture potential lifetime benefits and costs associated with a UCI. The model employed a six-month cycle length and allowed for death from all-cause mortality (which increased in likelihood as the cohort aged) to be incorporated as an absorbing state.

Parameter estimates were incorporated as mean values with an associated prior distribution representing uncertainty surrounding the mean. Distributions were sourced from the literature, along with expert opinion where data on distributions were not available. Assumptions on distributions were made for those remaining parameter estimates, based on the family to which the parameter belonged.

The discount rate for future QALYs and costs was 3%, with a 1.5% discount rate tested within a sensitivity analysis according to the Ministry of Social Affairs and Health Pharmaceuticals Pricing Board [17]. A discount rate of 5% was also tested within the sensitivity analysis to help compare results between the UK and Sweden, as recommended by The Panel on Cost-effectiveness in Health and Medicine [18]. All costs were expressed in 2021 Euro prices.

### Model structure

**Fig. 1:**
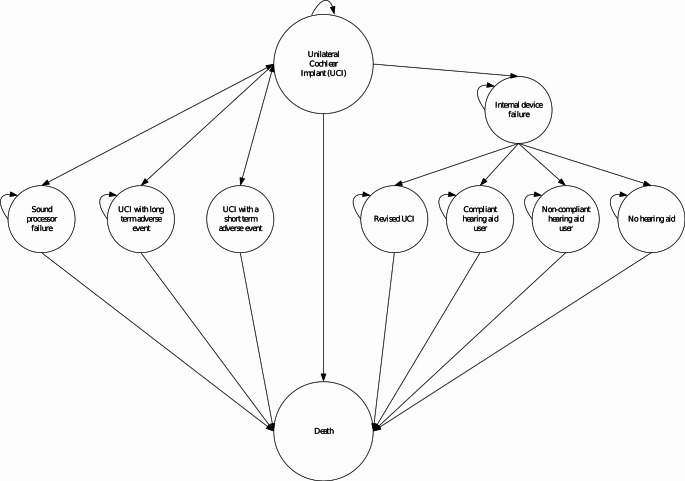
Markov model structure for unilateral cochlear implants

**Fig. 2:**
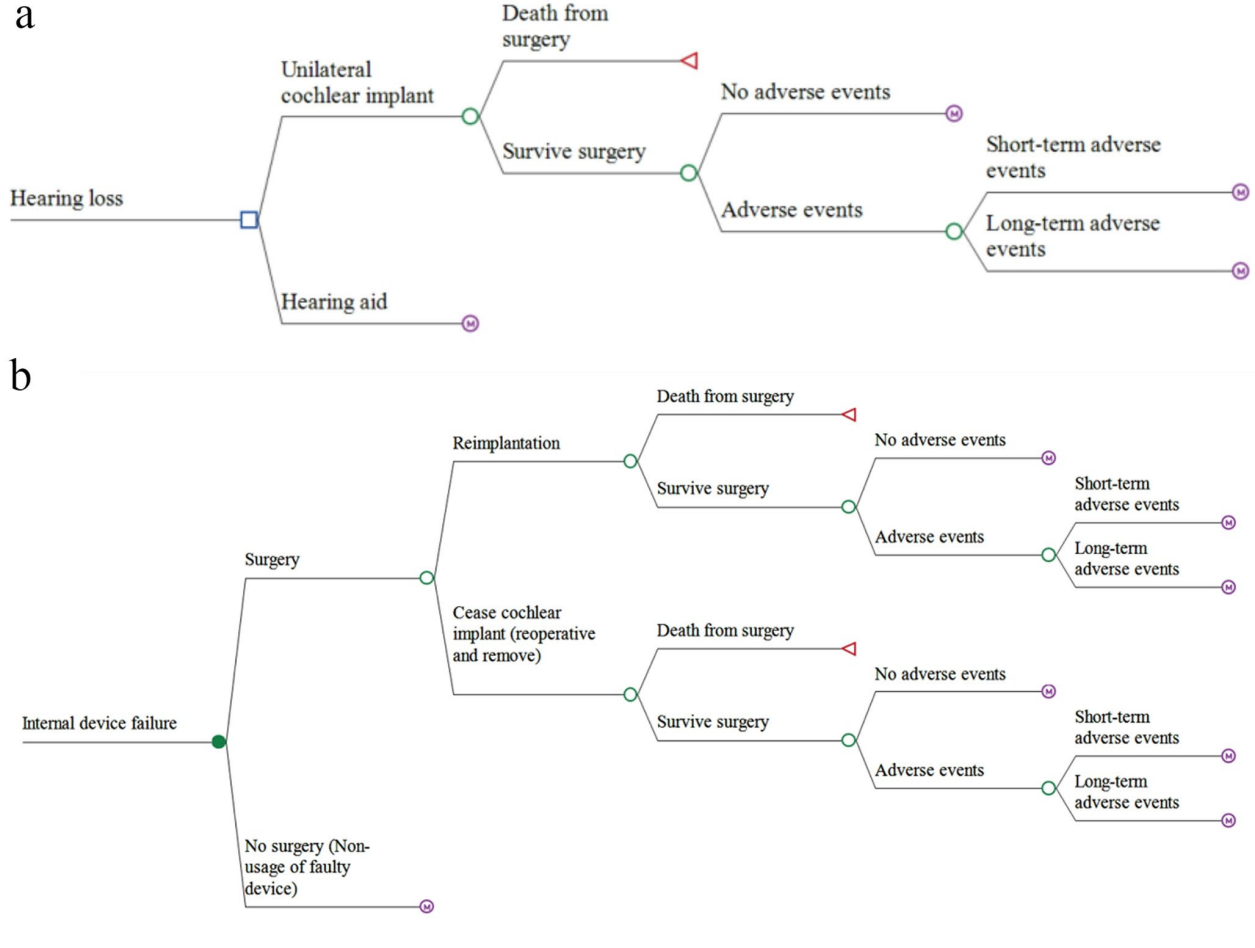
**a**: Treatment pathway in Finland for adults with severe to profound sensorineural hearing loss. **b**: Treatment pathway for patients experiencing and internal device failure. Note: While ‘Death from surgery’ was allowed for in the model, the probability of death from surgery was set to zero

The Markov model compared a UCI versus a hearing-aid (Fig. 1). The model incorporated several health states to capture the treatment pathway, potential UCI-related adverse events, device failures, and all-cause mortality (Fig. 2a and b).

The model structure allowed individuals who received a UCI to remain in their initial state (‘UCI’) or experience an adverse event or device failure. Adverse events were categorised as either short-term adverse events (e.g., dizziness, taste disturbance, tinnitus) that lasted one cycle (i.e., six months), or long-term events (e.g., vertigo) that lasted for their remaining lifetime.

Both internal device failure and sound processor failure could either be immediate (in the first cycle) following surgery or it could occur over time. The probability of internal device failure was estimated based on the risk of device failure and time from primary implantation to revision surgery from the literature. The probability of sound process failure was based on the Cochlear Reliability Report [19]. UCI users who experienced a sound processor failure received a replacement and approximately every nine years received an upgrade. It was assumed that there is no risk of death or adverse events related to sound processor failure.

Individuals who experienced an internal device failure may choose revision surgery and device replacement (Fig. 2b). These individuals may experience an adverse event for repeated surgery in the model, which could either be short-term or long-term. Alternatively, individuals who experience an internal device failure may also choose to remove the device or leave the faulty device in place.

Individuals who discontinue using a UCI have two options: they can either stop using their hearing aid altogether (‘No hearing aid’), or they can choose to continue using a hearing aid and may either be compliant (‘Compliant hearing aid user’) or non-compliant (‘Non-compliant hearing aid user’) with its use. ‘Death’ is an absorbing health state, accounting for all-cause mortality over time in the Finnish adult population.

### Model assumptions

Individuals were assumed to be diagnosed with severe to profound sensorineural hearing loss in both ears. Individuals were using hearing aids in both ears prior to receiving a UCI. All persons deemed eligible for implantation were assumed to receive a UCI (i.e., no persons drop off the waiting list before surgery). Any future hearing loss the individual experiences does not impact health outcomes given hearing loss is already severe to profound.

Annual equipment maintenance costs start in the second year from receiving a UCI. The internal component lasts a lifetime unless device failure occurs. It is assumed that the internal part of the CI system or its magnet does not have to be removed prior to MRI imaging or radiotherapy.

To ensure a parsimonious model, it was assumed that a sound processor upgrade would not result in additional health benefits, nor would receiving a UCI impact the prevalence or severity of other health conditions (e.g., depression or dementia). Both assumptions are likely conservative as processor upgrades often improve usability and possibly hearing performance.

Other assumptions were that CI surgery is never abandoned during the procedure, the probability of death from surgery is zero, all initial CIs and re-implants are successful, and that an implanted CI does not impact life expectancy.

### Model inputs

Model inputs and their sources are provided in Table 1. Data were initially identified through literature and reflect inputs included in studies exploring the cost-effectiveness of UCI in the UK and Sweden [15, 16]. Differences between the current and earlier studies included the annual discount rate to reflect guidance provided by the Ministry of Social Affairs and Health Pharmaceuticals Pricing Board in Finland, the average age of UCI receipt in Finland, and the proportion of patients deemed eligible for a CI after initial assessment based on opinion from experts in Finland [17]. This study also revised the probability of an internal device failure based on clinical recommendations and external sound processor failure rates according to the Cochlear Implant Reliability Report [19].

**Table 1:** Model parameters

Parameter	Value	Source	95% Confidence interval or range	Distribution
Time horizon	Lifetime	Authors assumption based on the expected benefits and costs of a cochlear implant	NA	NA
Annual discount rate	3%	Ministry of Social Affairs and Health Pharmaceuticals Pricing Board, 2024 [17]	1.5%, 5%	Assumed certain
Average age	62 years	Dietz et al., 2022 [33]	28, 88 years	Gamma
Proportion of people deemed eligible for a cochlear implant after initial assessment	0.95	Expert opinion	0.89, 0.99	Beta
Probability of a cochlear implant internal device failure	0.028	Wang et al., 2014 [38]	0.021, 0.037	Beta
Annual probability of an electrode migration requiring surgery for first two years after receiving a UCI	0.0025	Dietz et al., 2016 [39]	0.00012, 0.00840	Beta
Annual probability of a sound processor failure	0.0005	Cochlear Reliability Report 2022 [19]	0.0000, 0.0025	Beta
Probability a patient elects to discontinue using their cochlear implant	0.077	Kumar et al., 2016 [40]	0.009, 0.206	Beta
Proportion of people who have severe to profound hearing loss who are hearing aid compliant	0.50	Bond et al., 2009 [41]	0.061, 0.939	Beta
Mean lifetime of an acoustic hearing aid	5 years	Bond et al., 2009 [41]	1.4 years, 11.0 years	Gamma
Mean time to sound processor upgrade	106 months	Cochlear Limited Internal Database (2022)	29 months, 232 months	Gamma

### Utilities

A systematic literature review was undertaken to extract health state utility values relating to hearing loss, CIs and hearing aids in adults aged 18 years and over. There were no studies from Finland so studies from other countries were explored. Utilities derived from persons with severe to profound sensorineural hearing loss within a randomised controlled trial were prioritised, along with studies using the Health Utilities Index 3 (HUI3), given this instrument includes hearing and speech domains [20–22].

The utility increment associated with receiving a CI (0.210) was based on a prospective longitudinal study of 38 adults with profound post-lingual hearing impairment in Sweden [23]. This was added to the utility decrements from severe to profound sensorineural hearing loss prior to a CI, which was estimated by subtracting the pre-CI HUI3 utility score from Bergman et al. (2020) to the Finnish HUI3 population utility norms (Table 2) [23, 24].

**Table 2:** Utility, disutility and probability of occurrence for health States and adverse events

Parameter	Value	Source	Confidence interval or range	Distribution
**Health state**				
Utility of severe and profound HL prior to a CI	0.450	Bergman et al. (2020) [23]	0.426, 0.475	Gamma
Utility decrement from population utility norms for persons with severe and profound HL	0.391	Calculated by subtracting the HUI3 utility score prior to a cochlear implant from the Finnish HUI3 population utility norm (Official Statistics of Finland, 2023) [24]	NA	NA
Utility increment associated with receiving a UCI	0.210	Bergman et al. (2020) [23]	0.186, 0.236	Gamma
**Short term adverse events**				
Dysgeusia (taste disturbances)	Mean disutility 0.020	Expert opinion	0.005, 0.044	Gamma
	Probability 0.065	Inverse weighted average of (Hansen et al., 2010) [42] (Jeppesen and Faber, 2013) [43] (Farinetti et al., 2014) [44]	0.0649, 0.0652	Beta
Vertigo	Mean disutility 0.033	Swan (2011) [45]	0.009, 0.072	Gamma
	Probability 0.1937	Inverse weighted average of (Hansen et al., 2010) [42] (Jeppesen and Faber, 2013) [43] (Farinetti et al., 2014) [44] (Venail et al., 2008) [46]	0.0454, 0.4151	Beta
Infection	Mean disutility 0.042	Prosser (2004) [47]	0.011, 0.092	Gamma
	Probability 0.01542	Inverse weighted average of (Hansen et al., 2010) [42] (Jeppesen and Faber, 2013) [43] (Stamatiou et al., 2011) [48] (Farinetti et al., 2014) [44] (Venail et al., 2008) [46]	0.01539, 0.01545	Beta
Tinnitus	Mean disutility 0.050	Happich (2009) [49]	0.014, 0.110	Gamma
	Probability 0.0357	Inverse weighted average of (Jeppesen and Faber, 2013) [43] (Farinetti et al., 2014) [44] (Venail et al., 2008) [46]	0.0356, 0.0358	Beta
**Long term adverse events**				
Vertigo	Mean disutility 0.033	Swan (2011) [45]	0.009, 0.072	Gamma
	Probability 0.014	Inverse weighted average of (Hansen et al., 2010) [42] (Jeppesen and Faber, 2013) [43]	NA	NA

The literature identified 17 different types of adverse events associated with CIs, excluding device complications such as electrode migration. Only those with a prevalence of greater than 1% were included in the model, which included dysgeusia (taste disturbance), vertigo, tinnitus and wound infection. It was assumed that short-term adverse events would last for six months. They were included in the model as a weighted average of disutility, based on a variety of sources from the published literature, using the probability of experiencing an adverse event (Table 2).

### Resource use

**Fig. 3:**
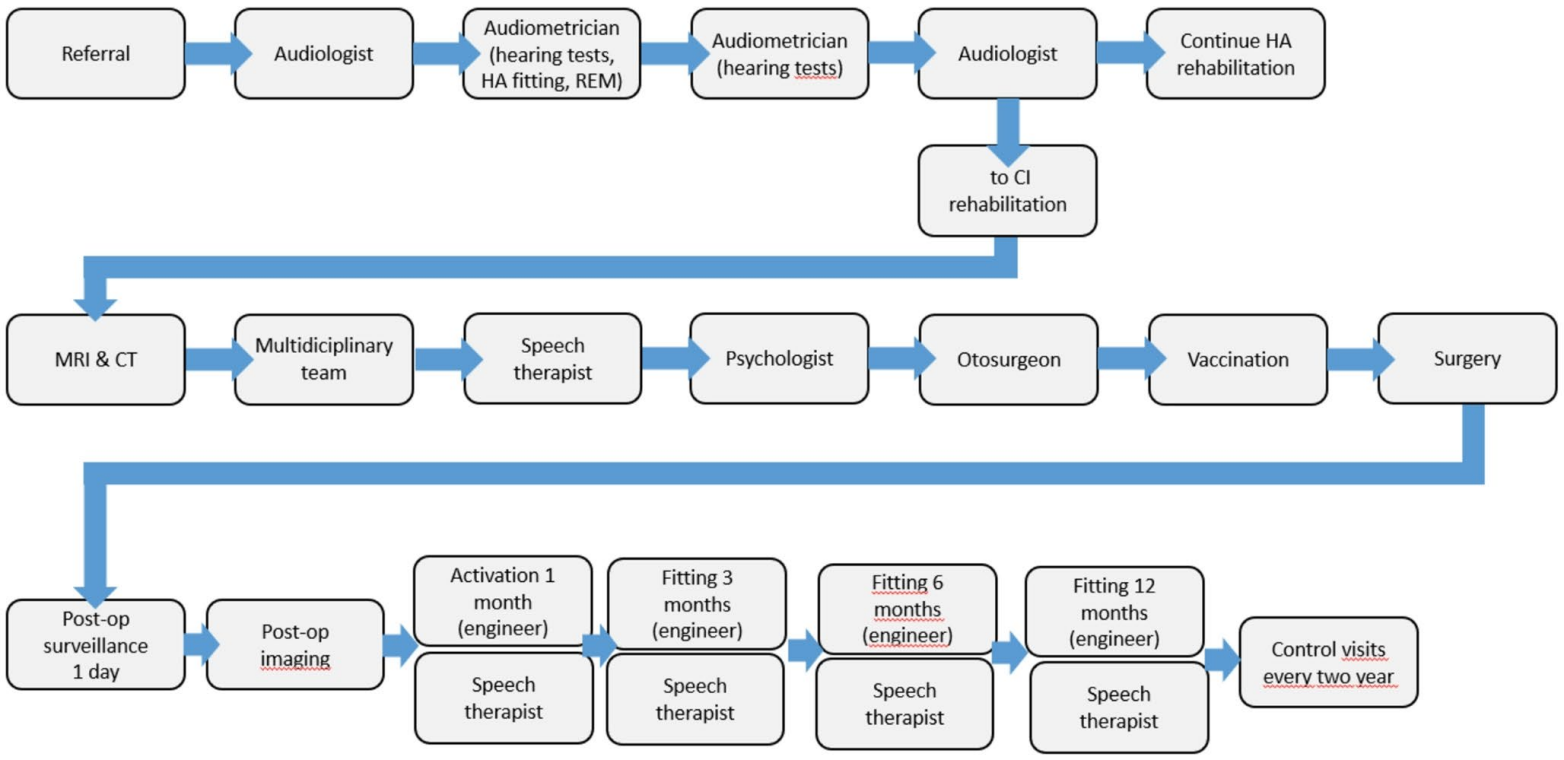
Average treatment pathway in Finland

Resource use was derived from consulting with the Oulu, Turku and Kuopio University Hospitals in Finland on their clinical pathways towards receiving a UCI. This information was used to develop an average treatment pathway a patient is expected to take in Finland to receive a UCI, from receiving an initial referral for the evaluation of UCI (Fig. 3). Resource use was divided into pre-implant assessment, surgery (the implant device and surgery), device programming and maintenance including patient follow-up, sound processor replacement / upgrade, explants and re-implants, and complications (short- and long-term adverse events).

### Costs

Costs were calculated by multiplying the volume of resource use associated with receiving a UCI by their unit costs (Supplementary Table 1). Resource use was based on clinical expert opinion sought within the development of the clinical pathway. Hearing aid costs were based on the number of hearing aids a person was using before receiving a UCI. Individuals who receive a UCI were assumed to no longer require a hearing aid in the ear receiving the UCI (unless they choose to remove the UCI due to internal device failure) and continue wearing their contralateral hearing aid. Unit costs were averaged from the list prices of each of the five Finnish University Hospitals. Average Diagnostic-related group (DRG) reimbursement rates were used to value inpatient costs (operation and aftercare) in tertiary care. The device costs were the same for each institution.

### Sensitivity analysis

An analysis was undertaken to assess the sensitivity of the ICERs to key model parameters. Parameters were chosen based on their expected level of uncertainty and potential influence on ICERs. Their range was dictated by the 95% confidence interval around the estimate (where available), while an arbitrary range was determined for all other parameters [25]. Parameters on resource use were assumed to follow a gamma distribution and unit costs were assumed to be certain.

A probabilistic analysis using Monte Carlo simulation was also conducted, using distributions attached to each model parameter, with 10,000 samples drawn at random from these distributions to calculate a distribution of ICERs. Fixed random number seeds were used to minimise random simulation error [26]. A cost-effectiveness acceptability curve (CEAC) was calculated to estimate the probability of the ICER being cost-effective across the assumed cost-effectiveness threshold of €25,000 per QALY gained [25].

### Model validation

Model validation was undertaken using the Assessment of the Validation Status of Health-Economic decision models (AdViSHE) tool [27]. Internal consistency was checked by ensuring probabilities lay between zero and one and probabilities following a chance node summed to one. Two additional researchers reviewed the mathematical logic of the model, including the equations, coding and model inputs. Null and extreme values were used within the sensitivity analysis to determine whether subsequent results met a priori expectations. The model was validated by clinical experts on whether the assumptions and structure of the model were reliable and could be reasonably explained.

## Results

### Base case results

A UCI for someone who had previously worn a hearing aid in the ear that received the CI led to an average incremental lifetime cost increase of €43,189 and an additional 2.97 QALYs. This equated to an ICER of €14,528 per QALY gained (Table 3).

**Table 3:** Cost-effectiveness results

	Cost (€)	QALY	ICER (€ per QALY gained)
**Deterministic results**			
Hearing aid	717	5.26	−
Unilateral cochlear implant	43,905	8.23	−
Difference	43,189	2.97	14,528
**Probabilistic results** ^**1**^			
Mean incremental difference	43,066	3.03	14,278
Confidence interval (2.5%)	27,676	2.65	8,728
Confidence interval (97.5%)	71,589	3.47	23,690

### Sensitivity analysis

**Fig. 4:**
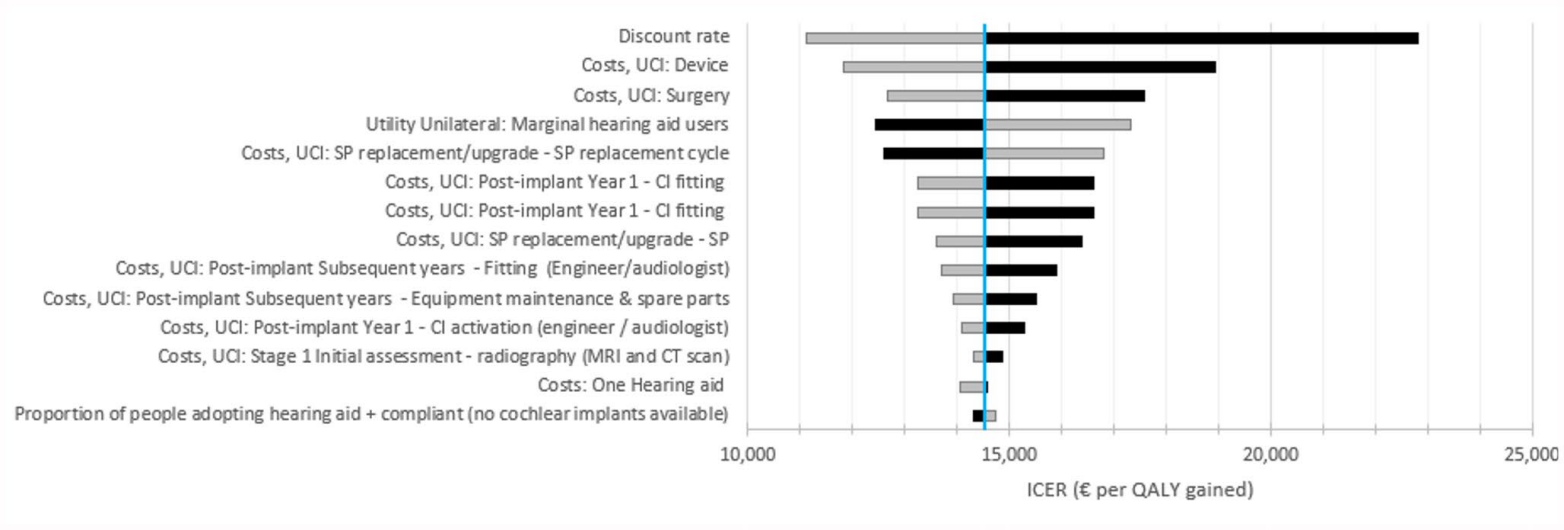
Sensitivity of the ICER for a unilateral cochlear implant vs. a hearing aid. Note: Sensitivity of the ICER associated with UCIs for people that had previously worn a hearing aid. The grey bar represents the variable going down, while the black bar represents the variable increasing

The ICER associated with a UCI compared to a hearing aid was most sensitive to the discount rate, device and surgery costs for CI, utility increment from UCI treatment, costs associated with CI fitting, and the cost and upgrade cycle of sound processors (Fig. 4). The ICER was also sensitive to the age upon which a person receives a UCI, with a lower ICER (more cost-effective) associated with implants at a younger age, conditional on the same hearing loss (Supplementary Fig. 1).

**Fig. 5:**
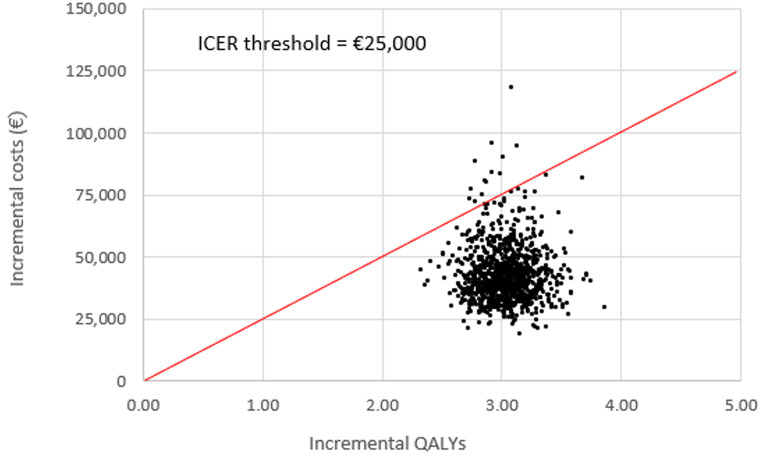
Mean differences in costs and QALYs (unilateral cochlear implant vs. hearing aid). Note: Derived from Monte Carlo simulation using 10,000 iterations

All simulated results for a UCI in the probabilistic sensitivity analysis (PSA) fell into the north-east quadrant of the cost-effectiveness plane, indicating that a UCI was more expensive but also more effective than a hearing aid (Fig. 5). Most simulated mean differences in costs and QALYs were below the €25,000 per QALY gained threshold, indicating a UCI compared to a hearing aid is likely to be cost-effective. The CEAC suggests that a UCI had a 98.5% likelihood of being cost-effective when compared to a hearing aid using a €25,000 per QALY gained threshold (Supplementary Fig. 2).

## Discussion

Compared to a hearing aid, a UCI is a relatively expensive treatment for hearing loss, and it also requires life-long aftercare including regular check-ups, software and sound processor upgrades and sometimes a device replacement. Since clinical pathways vary, along with patient selection and reimbursement between different countries, cost-effectiveness analyses for different health care settings are warranted. Additionally, as cochlear implantation continues to evolve in terms of its indications and clinical pathways, and as it has become a standard therapy in our increasingly aging populations, a growing number of patients are becoming eligible for this treatment. Consequently, institutions are forced to adapt their clinical pathways to manage this surge in demand efficiently. Given these significant changes, it is imperative that cost-effectiveness analyses are updated to accurately reflect the current clinical protocols.

The present study is the first to evaluate the cost-effectiveness of UCI in Finnish adults. The results suggest that a UCI in patients with severe to profound sensorineural HL is cost-effective. For the Finnish reference patient with UCI aged 62 (mean) years who had little benefit from prior hearing aid(s), the average incremental lifetime cost was €43,189 with an additional 2.97 in QALYs; this equated to an ICER of €14,528 per QALY gained.

Although willingness-to-pay thresholds are not formally applied in the Finnish health care system, the estimated ICER is below the indicatory cost-effectiveness threshold of NICE. Our results are in line with the NICE Health Technology Assessment Report conducted in 2009 and with a recent updated independent report which accounted for increased healthcare unit costs [11, 16].

In the 2009 NICE report the ICER was £14,163/QALY (approx. €16,000/QALY) for adults aged 50 years with a 100% probability of being cost-effective applying the £20,000/QALY NICE threshold. The ICERs reported for the UK in the updated analyses were lower; £11,946/QALY (approx. €14,000/QALY) and £10,499/QALY (approx. €12,000/QALY) with a 93% and 99% likelihood of being cost-effective for a UCI when compared to hearing aid or no hearing aid, respectively [16].

Higher ICER estimates (approx. €26,000) have been reported by the Swedish National Board of Health and Welfare for Swedish adult UCI recipients [28]. However, Swedish clinical practice and costs have changed since that study was completed and an updated study that considered these changes demonstrated similar ICERs and CEACs as in this study for Finland [15].

Our results align with recent UK and Swedish studies on the cost-effectiveness of UCIs [15, 16]. Despite differences in indications, clinical pathways and device unit costs, the ICERs ranged from around €12,000 to €15,000 per QALY gained (Supplementary Table 2).

Across the three countries, the incremental lifetime cost and QALYs were highest for UCI recipients versus hearing-aid recipients in the UK, likely because of the higher device costs and younger age at implantation (mean 52.8 years in the UK compared with 62 and 61 years in Finland and Sweden, respectively).

We applied the same select model inputs from the Finish model to the Swedish and UK models where there were differences to estimate the impact on ICERs for these two countries (Supplementary Table 2). Applying the same mean age at implantation in the Finnish model to the UK model increased the UK model ICER by 19%. Decreasing the discount rate from 3.5 to 3.0% in the UK model decreased the UK ICER by 13%. Using the same utility increment 0.21 after UCI in the UK model as in the Finnish and Swedish models decreased the UK ICER by 20% [23].

Across all three studies, the Finnish model reported the second greatest nominal incremental cost but the smallest incremental QALY, resulting in the slightly higher ICER. However, this result should be interpreted with caution. There is some uncertainty in the results so the difference in ICERs between the UK, Sweden and Finland is unlikely to be statistically significant. Unit costs were also presented in different years across each study, so healthcare cost inflation would have increased the unit costs for Sweden and Finland relative to the UK model.

While the structure of the UK, Swedish and Finnish models are virtually the same, small differences have impacted the ICERs. For example, based on clinical expert advice, the Finnish model incorporated the costs and disutility associated with surgical complications more comprehensively, including e.g., electrode migration, an underreported adverse event not considered in the UK and Swedish models. This would have increased the ICER within the Finnish model. Differences in age at implementation and expected aged of death based on life tables across the three models also impacted the QALYs gained.

### Age

While cost effectiveness analysis has been criticized for creating inequitable outcomes they allow for standardized comparisons of different therapies and procedures across different specialties, which can serve as a basis for making funding decisions.

The cost-effectiveness of UCIs depended on the remaining life expectancy of the recipient as the QALY is estimated by the size of the health improvement and the duration of the improvement. Despite increased costs associated with receiving a UCI earlier in life, such as additional processor upgrades, the associated increase in health benefits more than compensate from a cost-effectiveness perspective.

The average age of Finnish UCI recipients at the time of intervention within the model was 62 years based on clinical data, compared to 61 years for Sweden and 53 years for the UK. If the average age of implementation in Finland was equivalent to the UK, while holding all other inputs constant, the average ICER for UCIs among Finnish adults would reduce (become more cost-effective) by 15%.

Scenario analysis indicated that implanting a UCI at a younger age, conditional on the same amount of hearing loss, would result in a significant decrease in the ICER, however this is not a linear relationship, instead reflecting an exponential relationship (Supplementary Fig. 1). Consequently, the marginal reduction in the ICER from receiving a UCI will become less as the recipient becomes one year younger, and the marginal increase in the ICER becomes more as the recipient becomes one year older. This suggests that for people with severe to profound hearing loss, it is more cost-effective to implant a UCI earlier in their life.

Since sensorineural HL is a progressive chronic condition, earlier intervention may not only be beneficial from a health economics perspective, but also offers substantial benefits, particularly in terms of increased social engagement, preservation of auditory pathways, and prevention of cognitive decline. Early implantation helps to preserve the brain’s auditory pathways, which can deteriorate with prolonged auditory deprivation, supporting better long-term auditory processing with more favorable hearing outcomes with CIs [29–31]. In addition, adopting a cochlear implant and its technology is easier when patients are younger [32].

### Strengths and limitations

This study relies on a mathematical model requiring a set of assumptions derived from the literature and from expert opinion. The unit cost estimates were collected from institutional data reporting average costs. They may not reflect the true costs of implanting a UCI for a specific hospital given there are likely variations in costs, however using average unit costs is likely to better reflect the cost-effectiveness of a UCI at a system level.

There was also some uncertainty within model inputs. Utility increment estimates were based on a study conducted in Sweden, since no data that estimates utility increments are available for Finnish UCI recipients [23]. However, this limitation is not expected to have a significant impact on the ICER. A recent study reported comparable improvements in patient-reported outcome measures for Finnish UCI recipients as reported elsewhere [33]. Therefore, it is plausible to assume the utility increments are also similar.

Within the model, “no intervention” for hearing loss was considered to result in only the cost of purchasing hearing aids excluding its life-long aftercare. Costs derived from the increased risk of untreated hearing loss for depression, social isolation, loss of independence, cognitive decline or even dementia were not considered. Several studies have found up to 46% higher overall health care costs among persons with untreated hearing loss compared to matched persons without hearing loss [34, 35].

Additionally, non-health related costs of untreated hearing loss, such as lost productivity and lost opportunities in education, were also not included in the model. Untreated hearing loss is estimated to impose a substantial economic cost of around $981 billion annually [36]. Improving coverage of hearing management to 90% within a decade, including increased use of cochlear implants, would yield $15 in benefits for every $1 invested and result in health gains worth more than $3.3 trillion [37]. Thus, the cost-effectiveness analyses in the present study likely underestimate the utility increment, and cochlear implants would be even more cost-effective if these additional factors could be included in the model.

## Conclusion

UCIs in Finnish adults with hearing loss and with no or marginal benefits of hearing aid(s) is cost-effective, becoming more cost-effective the younger a person receives the UCI. The ICER compared to those currently estimated in the UK and Sweden were similar, despite differences in unit costs and clinical pathways. Future research is required to update utility estimates that reflect local care and advancements in CI technology. In addition, future models should assess the costs and benefits of UCI more comprehensively by also considering the medical and non-health-related risks associated with untreated hearing loss.

## Electronic supplementary material

Below is the link to the electronic supplementary material.

Supplementary Material 1

## Data Availability

Data used in this study has been previously published and Finnish healthcare data are openly accessible.
