# Supplementary material for: The cost-effectiveness of unilateral cochlear implantation in the Finnish health care
Source: Eur Arch Otorhinolaryngol. 2025 Jun 6;282(11):5551–62. doi: 10.1007/s00405-025-09468-9 (PMC12605616; doi:10.1007/s00405-025-09468-9)

**Supplementary Table 1: Resource use and unit costs in Finland**

| **Item** | **No. of visits** | **Units** | **Unit type** | **Unit cost (€)** |
| --- | --- | --- | --- | --- |
| **Primary cochlear implantation** |  |  |  |  |
| **Pre-surgery** |  |  |  |  |
| **Referral** |  |  |  |  |
| Hospital audiologist | 1 | 1 | Consultation | 280 |
| Audiogram and speech audiometric tests |  | 1 | Test | 120 |
| Promontorium test | 0.1 | 1 | Test | 100 |
| **Stage 1: Initial assessment and testing** |  |  |  |  |
| CI unit audiologist | 1 | 1 | Visit | 280 |
| Audiological assessment | 1 | 1 | Visit | 150 |
| MRI and CT scan |  | 1 | Unit | 832 |
| **Stage 2: Medical assessment** |  |  |  |  |
| Multidisciplinary team meeting (audiology, SLT, ENT specialist) | 1 | 1 | Visit | 455 |
| Speech therapist | 1 | 1 | Visit | 256 |
| Psychologist | 1 | 1 | Visit | 256 |
| Otosurgeon | 1 | 1 | Visit | 150 |
| Anaesthetist consultation | 0.1 | 1 | Unit | 200 |
| Meningitis vaccination |  | 1 | Unit | 80 |
| **Hospital Ward** |  |  |  |  |
| Unilateral CI operation – Surgical cost | 1 | 1 | Surgery | 7,578 |
| Unilateral CI operation – Internal device and sound processor cost |  | 1 | Implant | 11,014 |
| **Hearing aids** |  |  |  |  |
| One hearing aid (including fitting) | 1 | 1 | Unit | 695 |
| Pair of hearing aids (including fitting) | 1 | 1 | visit including hearing aids (bilateral) | 995 |
| **Post-implantation** |  |  |  |  |
| **Initial care – Year 1** |  |  |  |  |
| Post operative CT imaging | 1 | 1 | Unit | 177 |
| CI activation (engineer/audiologist) | 1 | 1 | Visit | 471 |
| CI fitting |  |  |  |  |
| 1 week fitting (engineer and audiologist) | 1 | 1 | Visit | 525 |
| 1 month fitting (engineer and speech therapist) |  | 1 | Visit | 525 |
| 3 months fitting (engineer and speech therapist) | 1 | 1 | Visit | 525 |
| 6 months fitting (engineer/audiologist) | 1 | 1 | Visit | 525 |
| 12 months fitting (engineer/audiologist and speech therapist) | 1 | 1 | Visit | 525 |
| **Follow-up care (Year 2 and beyond)** | | | | |
| Fitting (Engineer/audiologist), per year | 1 | 1 | - | 280 |
| Annual equipment maintenance and spare parts |  | 1 | Unit | 200 |
| CI annual administration | 1 | 1 | Hour | 50 |
| **Sound processor replacement/upgrade** |  |  |  |  |
| External component (sound processor) | 1 | 1 | Unit | 5,762 |
| Audiologist (fitting and tuning visit) | 1 | 1 | Visit | 280 |
| **Internal device failure** |  |  |  |  |
| **Explant** |  |  |  |  |
| Surgical cost |  | 1 | Surgery | 3,950 |
| Audiologist (assessment) |  | 1 | Hour | 280 |
| **Re-implantation** | | | | |
| Audiologist pre-operative assessment | 1 | 1 | Visit | 280 |
| Otosurgeon consultation | 1 | 0.5 | Visit | 150 |
| Unilateral CI operation – Surgical cost | 1 | 1 | Surgery | 7,578 |
| Unilateral CI Operation - Internal device cost | 1 | 1 | Unit | 5,420 |
| **Electrode migration** |  |  |  |  |
| Re-surgery |  | 1 | Surgery | 4,500 |
| **Short-term adverse events** |  |  |  |  |
| Infection |  |  |  |  |
| GP visit | 1 | 1 | Consultation | 150 |
| Antibiotics |  | 1 | Course | 150 |
| **Long-term adverse events** |  |  |  |  |
| Vertigo |  |  |  |  |
| Neuro-otologist |  | 1 | Consultation | 280 |

Note 1: Assuming nominal conversion rates of SEK 1 = € 0.095 and £ 1 = €1.15. Note 2: Assuming cost-effectiveness threshold to be €25,000 in Finland, £20,000 in the UK and SEK250,000 in Sweden.

Supplementary Table 2: Cost-effectiveness results (UCI versus hearing aid)

|  | **Finland**  **(current study,**  **2023 prices)** | **UK**  **(Cutler 2022,**  **2018 prices)** | **Sweden**  **(Gumbie 2021,**  **2019 prices)** |
| --- | --- | --- | --- |
| **Selected input parameters** |  |  |  |
| Age at implantation (years) | 62 | 52.8 | 61 |
| Discount rate, costs and QALYs | 3% | 3.5% | 3.0% |
| Proportion eligible for Cochlear after triage 0.95 | 0.95 | 0.70 | 0.56 |
| Electrode migration requiring re-surgery (no replacement) | 0.5% first 2 years | Not considered | Not considered |
| Utility decrement from severe/profound HL | 0.391 | 0.373 | 0.391 |
| Utility increment after UCI | 0.21 | 0.151 | 0.21 |
| **Base case** |  |  |  |
| Incremental cost | €43,189 | £37,988  (€43,662) | SEK435,147  (€36,939) |
| Incremental QALY | 2.97 | 3.18 | 3.10 |
| ICER (per QALY gained) | €14,528 | £11,946  (€13,737) | SEK140,474  (€11,925) |
| Likelihood of being cost-effective (Note) | 98.5% | 93.0% | 92.2% |
| **Sensitivity analyses** |  |  |  |
| Age at implantation: 62 years | €14,528 | €16,3047 (19%) | €12,172 (2%) |
| Discount rate 3.0% for costs and QALYs | €14,528 | €11,949 (−13%) | €11,925 (0%) |
| Proportion eligible for Cochlear after triage 0.95 | €14,528 | €13,674 (0%) | €11,305 (−5%) |
| Utility decrement from severe/profound HL 0.391 | €14,528 | €14,416 (5%) | €11,925 (0%) |
| Utility increment after UCI 0.21 | €14,528 | €10,533 (−23%) | €11,925 (0%) |
| Age at implantation 62 years and discount rate 3.0% for costs and QALYs | €14,528 | €14,698 (7%) | €12,172 (2%) |
| Utility decrement from severe/profound HL 0.391 and utility increment after UCI 0.21 | €14,528 | €10,932 (−20%) | €11,925 (0%) |

Assuming nominal conversion rates of SEK 1 = € 0.095 and £ 1 = €1.15.

Note: Assuming cost-effectiveness threshold to be €25,000 in Finland, £20,000 in the UK and SEK250,000 in Sweden.

Supplementary Figure 1: Variation of the ICER by age of cochlear recipients

**
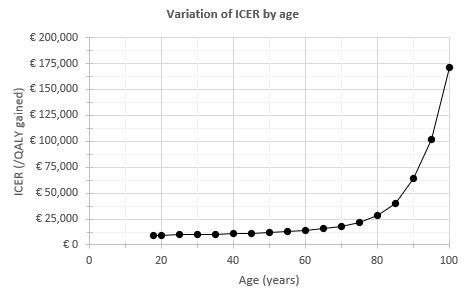
**

Supplementary Figure 2: Cost-effectiveness acceptability curve (UCI vs hearing aid)


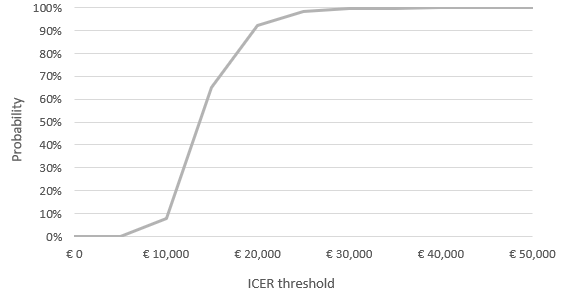

Supplement: Supplementary file 1 — Supplementary Material 1 [file 405_2025_9468_MOESM1_ESM.docx]
